# TARGIT-E(lderly)—Prospective phase II study of intraoperative radiotherapy (IORT) in elderly patients with small breast cancer

**DOI:** 10.1186/1471-2407-12-171

**Published:** 2012-05-08

**Authors:** Christian Neumaier, Sperk Elena, Welzel Grit, Abo-Madyan Yasser, Kraus-Tiefenbacher Uta, Keller Anke, Gerhardt Axel, Sütterlin Marc, Wenz Frederik

**Affiliations:** 1 Department of Radiation Oncology, University Medical Centre Mannheim, University of Heidelberg, Theodor-Kutzer-Ufer 1-3, Mannheim, 68167, Germany; 2 Department of Radiation Oncology and Nuclear Medicine (NEMROCK) Faculty of Medicine, Cairo University, Cairo, Egypt; 3 Department of Gynecology and Obstetrics, University Medical Centre Mannheim, University of Heidelberg, Theodor-Kutzer-Ufer 1-3, 68167, Mannheim, Germany

## Abstract

**Background:**

Patients ≥ 70 years with small, low-risk breast cancer who are operated but not irradiated how local relapse rates around 4% after 4 years. With adjuvant whole breast radiotherapy (WBRT) the local relapse rate drops to 1% after 4 years under Tamoxifen. It has been demonstrated that the efficacy of radiotherapy of the tumor bed only in a selected group can be non-inferior to WBRT.

**Methods/Design:**

This prospective, multicentric single arm phase II study is based on the protocol of the international TARGIT-A study. The TARGIT-E study should confirm the efficacy of a single dose of intraoperative radiotherapy (IORT) in a well selected group of elderly patients with small breast cancer and absence of risk factors. Patients will receive IORT (20 Gy with Intrabeam system/Carl Zeiss) during breast conserving surgery. In presence of risk factors postoperative WBRT will be added to complete the radiotherapeutic treatment according to international guidelines. Endpoints are the local relapse rate (within 2 cm of the tumor bed), ipsilateral in breast relapse, cancer-specific and overall survival and contralateral breast cancer as well as documentation of quality of life and cosmetic outcome.

The expected local relapse rates are 0.5/1/1.5% after 2.5/5/7.5 years, respectively. Discontinuation of the trial is scheduled if rates of local relapse rates rise to 3/4/6% after 2.5/5/7.5 years. Power calculations result in 540 patients with a calculated dropout rate of 20% and loss to follow-up of 20%, an alpha of 0.01 and a beta 0.05. There will be a pre- and a post-pathology stratum (n = 270 each).

**Discussion:**

It is a pragmatic trial in which each participating centre has the option to modify entry criteria and criteria for WBRT according to this core protocol after consultation with the steering committee and local ethics committee (e.g. size, free margins). Only centers with access to the Intrabeam system (Carl Zeiss) can recruit patients into the trial.

Its aim is to confirm the efficacy and toxicity of IORT in a well selected collective of elderly patients with breast cancer.

**Trail registration:**

NCT01299987

## Background

Randomized studies provide evidence that concerning the overall survival, breast conserving surgery (BCS) combined with postoperative radiotherapy is equally effective to modified radical mastectomy [1-3]. Postoperative radiation significantly decreases the local relapse rate [1,2] in comparison to BCS alone [3]. The recent metaanalysis of the Early Breast Cancer Trialists Collaborative Group (EBCTCG [3]) shows that the avoidance of 4 local relapses after 5 years can prevent one death in 15 years (4 to 1 rule).

Several studies have demonstrated in the past that complete omission of radiotherapy failed in terms of local tumor control. Fyles et al. [4] showed local relapse rates of 8% within a low risk group of patients with tumors of pathological stage T1/T2 and >50 years of age treated with BCS and antihormonal treatment (AHT). By adding postoperative whole breast radiation (WBRT) the rate of local relapse was reduced to 1% after 5 years. This effect was also seen in elderly patients with small tumors. Hughes et al. [5] observed low local relapse rates of 4% for patients treated with AHT only after BCS vs. 1% for patients with additional WBRT after 4 years, evaluating a low-risk group of patients with tumors < 2 cm and > 70 years of age. Apparently, all patients benefit from postoperative WBRT. However, due to the fact that up to 90% of all local relapses after breast conserving therapy are localized very close to the primary tumor, it might be possible to treat a selected group of patients with a relatively low risk of local recurrence with tumor bed irradiation only. Polgar et al. [6] provided evidence of the non-inferiority of accelerated partial breast irradiation (APBI) in comparison to WBRT in a small randomized trial. Very recently the TARGIT-A data [7] reported a non-inferior efficacy of tumor bed irradiation with single dose intraoperative radiation (Intrabeam system, Carl Zeiss Oberkochen) compared to WBRT regarding local relapse.

By using the Intrabeam system, the intraoperative radiotherapy (IORT) can be given during surgery with protection of the surrounding tissue while applying a biologically highly effective dose to the tissue adjacent to the tumor. Advantages of IORT are its high precision regarding beam application, possibility of protection of the skin and the prevention of tumor cell proliferation during the time interval between surgery and adjuvant WBRT and during fractionated WBRT. By manually positioning of the applicator in the tumor bed geographic miss is excluded and radiation exposure of risk structures like the heart and lung can be avoided. Therefore, it is possible to give a single high dose to the target while minimizing relevant side effects.

## Methods/Design

### Study design

This prospective, international, multicentric single arm phase II study is based on the protocol of the international TARGIT-A study. The TARGIT-E study should confirm the efficacy of a single dose of intraoperative radiotherapy (IORT) in a well selected group of elderly patients with small breast cancer and absence of risk factors. Patients treated with breast conserving surgery and IORT will only be followed by WBRT when risk factors are present. There will be a pre- and a post-pathology stratum.

### Pre-pathology stratum

Patients will enter the trial after careful clinical examination according to the inclusion criteria. Patients who are planned to have IORT at the time of primary surgery will be addressed in this stratum after giving consent prior to the planned surgery. Additional post-operative WBRT is given only when risk factors are present. This stratum will be closed after 270 patients are treated.

### Post-pathology stratum

Patients will be recruited for entry to the trial only once the pathological characteristics of the tumor have been reported. These patients will have local surgery as per usual practice and if the patient and the tumor fit in the criteria defined in the protocol and the investigator’s treatment policy, she will be asked for consent to enter the trial and if given she will have further surgery at which the wound is opened, the probe inserted and the radiation dose given. This stratum of patients addresses a much more explanatory question as their entry to the trial will be strictly defined on pathological grounds. However, the disadvantage for these patients is that they must have a second surgery. Including these patients will allow an assessment of the local control obtained in very good prognosis patients.

This strategy also allows for the entry of patients who have already received surgery at an outlying centre. The post-pathology stratum will recruit 270 patients.

### Trial design

**Figure 1:**
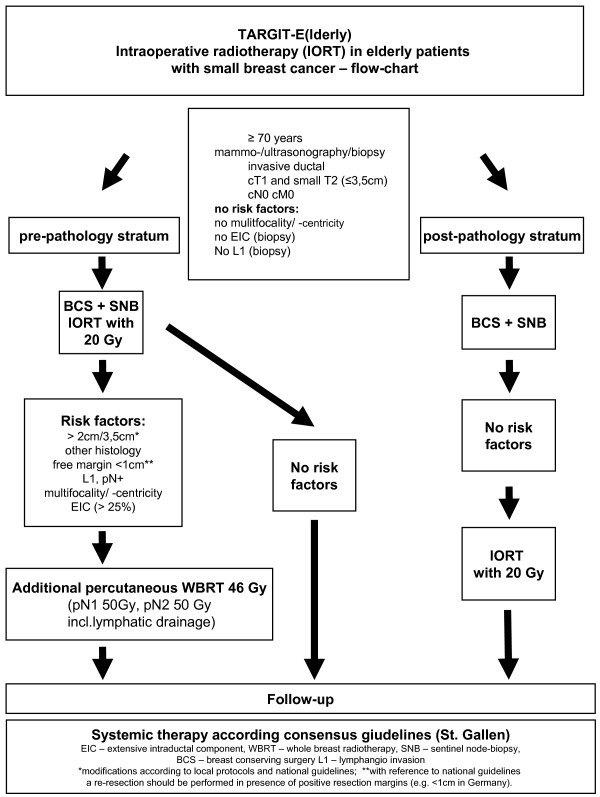
**Systemic therapy according consensus giudelines (St. Gallen).** EIC – extensive intraductal component, WBRT – whole breast radiotherapy, SNB – sentinel node-biopsy, BCS – breast conserving surgery L1 – lymphangio invasion *modifications according to local protocols and national guidelines; **with reference to national guidelines a re-resection should be performed in presence of positive resection margins (e.g. <1cm in Germany).

The treatment schedule of this single arm phase II study is shown in the study flow chart (Figure 1).

### Recruiting

first patient: January 2011

last patient: December 2015

follow-up: 10 years

### Study objectives

The objective of this single armed phase II study is to investigate the efficacy of a single intraoperative radiotherapy treatment within elderly low risk patients (≥ 70 years, cT1 and small cT2, cN0, cM0, invasive-ductal) which is followed by WBRT only when risk factors are present. For both primary and secondary efficacy measures descriptive statistics will be calculated and Kaplan-Meier plots will be generated. There will be a separate subgroup analysis of the pre- and post-pathology strata performed according to primary and secondary endpoints.

### Primary objectives

The primary objective is the rate of local relapse (within 2 cm around the tumor bed).

### Secondary objectives

The secondary objectives are ipsi- or contralateral breast cancer, cancer specific and overall survival, cosmetic outcome and quality of life.

## Patient selection

### Population

Elderly patients (≥ 70 years) with histologically verified unifocal (mammography, ultrasound), ductal-invasive, small (≤ 3.5 cm) breast cancer without clinical signs of lymph node involvement or distant metastases.

### Inclusion criteria

histologically verified invasive-ductal breast cancer

cT1 or small cT2 (≤ 3.5 cm) cN0 cM0

≥ 70 years of age

informed consent

compliance

### Exclusion criteria

extensive intraductal component (EIC)

multifocality/-centricity (mammography, breast ultrasound)

lymph vessel invasion (L1)

clinical signs of distant metastases or clinically suspicious lymph nodes

other histology

< 70 years

missing informed consent or non-compliance

bilateral breast cancer at the time of diagnosis

known BCRA1/2 gene mutations (genetic testing not required)

any exclusion criterion in the local centre’s treatment policy

positive resection margins (regarding the post-pathology arm)

## Treatment planning and dose prescription

### Radiologic diagnostics

All patients with primary breast cancer receive standardized staging.

Clinical examination, particularly breast and axilla palpation

Mammography and/or ultrasonography of the breast and histological verification of breast cancer

Breast magnetic resonance imaging is not required

### Surgery

All patients will have local excision of the primary tumor following appropriate clinical work-up. Surgery will be performed according to usual local practice with a complete excision of the tumor. The aim of the local excision should be to achieve a free margin of at least 10 mm whilst maintaining a good cosmetic outcome. The final histological margin should be at least 1 mm clear of all invasive and at least 2 mm for in-situ disease, otherwise a re-resection is recommended (may be modified according to local practice). For superficial tumors an ellipse of overlying skin should be excised to avoid overdosing of the skin. The depth of resection will depend on the position of the tumor within the breast and the size of the breast, but in many instances will extend to the pectoral fascia. In all patients, but especially in those women with non-palpable tumors with mammographic correlate in whom preoperative wire localization has been performed, the specimen should be well orientated with sutures or clips according to local protocols and x-rayed intraoperatively. The specimen x-ray should be examined in theatre to ensure complete excision of the lesion and to help with the assessment of adequacy of the margins. Further tissue should be taken (and marked) from a margin if the x-ray abnormality extends near the margin. It is recommended that intraoperative evaluation of the resection margin is performed by fast frozen sections by the pathologist. A standard sentinel node biopsy must be performed in all patients. Wound closure must be performed meticulously (air and water-tight) and sutures (if non-absorbable), should remain in place for 5–7 days.

### Radiotherapy—IORT dose prescription and delivery

The surgeon and radiation oncologist should choose the largest possible suitable applicator in order to ensure that the highest possible dose is delivered to the tumor bed tissue.

A dose of 20 Gy at the surface of the applicator (in water) is prescribed by the radiation oncologist and delivered to the breast tissue. This takes approximately 20–50 minutes, depending on the size of the applicator.

To minimize radiation induced side effects at the applicator surface and the skin, the distance should be more than 5 mm.

During the radiation treatment, the anesthesiologist, surgeon, radiation oncologist and physicist may remain in the room when necessary. To avoid unnecessary exposure, we recommend that as many people as possible leave the operating theatre, and those remaining either wear a leaded apron or remain behind a shielded screen.

### Conventional radiotherapy (WBRT)

If the final histopathological report provides evidence of risk factors as per local protocol (e.g. EIC, L1, mulitfocality/-centricitiy, larger diameter, other histology or too small free resection margins), a conventional postoperative percutaneous radiotherapeutic treatment with WBRT with 46 Gy will be added in 2 Gy per fraction. WBRT with 50 Gy will be performed in pN1 and WBRT including irradiation of the lymphatic drainage areas with 50 Gy in ≥pN2 situations. A delay of at least 5 weeks is required between IORT and initiation of WBRT. A delay of at least 14 days (21 days recommended) is required between chemotherapy and initiation of WBRT.

### Adjuvant treatment

Adjuvant systemic treatment will be performed according to international guidelines.

## Trial documentation, schedule and follow-up

### Documentation and follow-up

#### First Visit

Before treatment the general medical history report forms have to be filled out including the following items:

anamnesis (height, weight, general condition, Karnofsky index, medical history)

Localization of the tumor (mammography, ultrasound), if palpable (drawing)

histology (if already available)

quality of life assessment

- photo documentation (3 pictures, at 1 m distance—arms/hands on hips en face, and arms elevated en face + lateral from side of surgery)

#### Follow up

Follow-up visits will be scheduled after 6 weeks, 4.5 months, 6 months and annually after radiotherapy. Photographic documentation and assessment of quality of life will be performed at follow-up visits as well as documentation of general condition, weight, new symptoms according to CTC and LENT SOMA score. Furthermore mammography and ultrasound of the breast will be performed according to international guidelines.

Acute side effects in patients, who received postoperative WBRT, will be documented by the CTC score.

The follow-up and documentation of patient specific data will end after 10 years after therapy.

### Protocol deviation

Every protocol deviation has to be defined and documented for every patient.

The investigator has to consult the principle investigators, to discuss the type and amount of deviation and their possible consequences for the patient.

## Assessment of efficacy parameters

### Relapse

At each follow-up visit there will be a clinical examination to assess local relapse (within 2 cm around the tumor bed) by palpation. Relapses more than 2 cm distant from the tumor bed will count as an ipsilateral relapse. Therefore mammography and ultrasound of the breast will be performed according to international guidelines.

### Quality of life

To assess the quality of life two standardized, validated questionnaires of the EORTC (QLQ-C30 version 3.0 and BR23) will be used before IORT and documented during follow-up visits. The two questionnaires contain 53 questions and require about 10 minutes to be completed.

## Assessment of safety parameters

Classification and graduating of side effects are rated by “Common Toxicity Criteria” (CTC) of the National Cancer Institute (Bethesda/USA). If necessary photo documentation should capture acute toxicities to give suitable follow-up. The acquisition of late toxicities will be done based on the LENT SOMA score.

### Serious adverse events (SAE)

Serious adverse events (SAEs) are defined as any event that is fatal, life threatening, causes or prolongs hospitalization; causes disability or incapacity or requires medical intervention to prevent permanent impairment or damage, or any grade 4 toxicity.

In case of death an autopsy should be done to clarify the cause of death.

Regarding the comorbidity of elderly patients only radiation related SAEs (rrSAEs) grade 4 and 5 including date and on-/offset should be reported. Furthermore rrSAEs requiring therapy should be documented (e.g. antibiotics, aspiration).

## Statistical calculations for trial sample size

### Study hypothesis

This study is designed to confirm the non-inferiority of IORT in comparison to WBRT in elderly patients with low risk breast carcinoma.

Power calculations with an alpha set at 0.01, a beta set at 0.05 (power 95%), and an anticipated drop-out rate of 20% and a loss to follow-up of 20% result in 540 patients to be included. Calculations of the sample size were performed as described by A’Hern [8], Simon [9] and Fleming [10]. Each stratum has to recruit at least 270 patients.

### Description of the primary efficacy analysis and population

A single group of patients is studied. The proportion of patients who respond to treatment (i.e. local relapse free) will be used to estimate the true response rate with a 95% confidence interval. A response rate of 99.5%/99%/98.5% at 2.5/5/7.5 years is anticipated. A response rate of 97%/96%/94% is considered to be the minimum level.

Therefore, efficacy analyses will be performed at two stages.

### Planned interim analysis

The first after 144 patients and the second after 386 patients were treated. The expected local recurrence rate is 0.5/1/1.5% after 2.5/5/7.5 years, respectively. Stopping rules for the study are if the local recurrence rate is higher than 3/4/6% after 2.5/5/7.5 years. Therefore the efficacy of IORT will be rejected if more than 5 or > 8 patients show a local relapse, respectively.

An interim analysis of late breast toxicity is planned after a two-year follow-up of the first 144 patients. Results will be presented as frequency tables.

## Data handling, storage and archiving of date

### Data archiving

The responsible physician of the participating trial centre stores the vote of the ethics commission (copy), patient-identification list, patient informed consent and patient related data as well as the protocol in the Investigator’s Study File (ISF). During audits the primary data have to be accessible. After closing the trial, the ISF, all source data and documents have to be stored according to local legal regulation and ICH-GCP guidelines. The duration of storage in each centre is at least 30 years.

### Good clinical practice (GCP)

The physician is responsible that the accomplishment of the trial will be in concordance with the declaration of Helsinki, revised version of Somerset West, South Africa 1996 and the guidelines for good clinical praxis (ICH-GCP).

### Monitoring

To ensure quality, there will be an annual inspection at the trial centre sites by a study nurse or an investigator. Therefore, it will be obligatory to have insights in the trial investigation folder to evaluate the proper documentation and storage of the source data and CRFs according integrity, completeness of data and control of the dosing. A source data verification will be performed after recruiting the first three patients and will be annually checked by random samples.

### Audit

Trial centers, which do not have gathered experience with IORT during the TARGIT A trial will be inspected after 5 patients were treated by a physician and a physicist of the trial control center. This audit is to certify the correct application of IORT. Therefore, the responsible physician is obliged to present all trial related data to the auditors (see data archiving).

Auditors will schedule the appointment in order to be present when a patient will be treated according to the TARGIT-E protocol and to affirm the correct performance. Trial centers, which have already been audited during TARGIT-A, are excluded.

## Ethics, informed consent and safety

### Ethical aspects

This present study is subjected to supervision of the Ethics Commission of the Medical Faculty Mannheim, University of Heidelberg, Germany and the Bundesamt für Strahlenschutz (BfS).

The informed consent is performed written and verbally by the responsible physician. The patient has to have enough time (24 h) to decide whether she participates or not. During the education the physician has to indicate that a withdrawal is possible at each time and does not result in disadvantages for the patient.

### Safety

An interim analysis of late breast toxicity is planned after a two-year follow-up of the first 144 patients. Results will be presented as frequency tables.

### Discontinuation of the trial

#### Individual withdrawal

The treatment should be stopped, according to the following criteria:

Withdrawal of informed consent

Lack of compliance

Other required discontinuation of treatment due to insufficient skin-surface distance (< 5 mm) or unfavorable geometry will be decided by the physician.

The reasons for each withdrawal must be documented.

#### Investigator withdrawal

The steering committee is allowed to close the study ahead of schedule.

Other stop-criteria might be:

Insufficient recruitment

Unexpected high toxicity

Any grade V toxicity

More than 3 grade IV toxicities within the first 20 patients

More than 5 grade IV toxicities within the first 50 patients

Ethical and medical considerations with regard to new evidence (benefit/risk ratio).

More than 5 local relapses after the first safety report

## Disscussion

The TARGIT-A trial has shown the non-inferiority of partial breast radiation via the Intrabeam© system as an IORT compared to WBRT in patients with low risk breast carcinoma. This international, multicenter, prospective single arm study shall confirm the efficacy of IORT in elderly patients with low risk breast carcinoma.

## Abbreviations

IORT: Intraoperative radiotherapy; WBRT: Whole breast radiotherapy; BfS: Bundesamt für Strahlenschutz; CTC: Common toxicity score; EIC: Extensive intraductal component; SAE: Serious adverse effects; rrSAE: Radiation related serious adverse effects; ISF: Investigator study file.

## Competing interests

Carl Zeiss Surgical/Oberkochem/Germany supports radiobiological research at UMM.

## Author’s contribution

CN: writing, designing and processing (ethics and national authorities), patient selection, CRF design. EB: participates in design of the study, QoL definition of tools and endpoints. Grit Welzel: QoL definition of tools and endpoints, statistics, data management. YA-M & U K-T: patient selection, quality assurance for IORT. AK: Data-manager, CRF design, set-up of data bank, statistics. AG &MS: definition of surgical parts of the protocol, quality assurance for surgery, patient selection. FW: conceived the study, and participated in its design and coordination and helped to draft the manuscript, quality assurance IORT. All authors have approved the final version.

## Pre-publication history

The pre-publication history for this paper can be accessed here:

http://www.biomedcentral.com/1471-2407/12/171/prepub
